# The compliment sandwich

**DOI:** 10.7554/eLife.82928

**Published:** 2022-09-21

**Authors:** Eve Marder

**Affiliations:** 1 Volen Center and Biology Department, Brandeis University Waltham United States

**Keywords:** Living Science, scientific meetings, scientific disagreements

## Abstract

What is the best way to ensure that scientific criticism is heard and understood?

This summer many of us have returned to in-person scientific meetings with feelings of euphoria. At the first meeting I attended, almost every speaker commented that it was his or her first in-person meeting since the pandemic started, and how wonderful it was to see real people again. At that meeting, and the next one, I miraculously stayed awake during most, if not all, the talks, which for me is quite unusual. The talks were universally excellent, and I thought nothing of it when many of the audience commented, before asking a question, about how much they appreciated the talk.

Later in the summer, when in-person meetings were less of a novelty, I attended a Gordon Conference and was surprised that questions from the audience were still being prefaced with statements like “thanks so much for such an elegant and inspiring talk”. After this occurred twenty times in a row, I started wondering about my sanity. Admittedly, this meeting was only tangentially in my area, and my propensity to fall asleep during talks had returned, but I knew that while some of the talks were outstanding, others were less so.

Later, I mentioned this at lunch and was told that the questioners were using a “compliment sandwich”. For those who are not familiar with it, a compliment sandwich starts with praise, followed by a criticism or a difficult question, followed by more praise (or the same praise repeated). The purported advantage of this approach is that it softens negative feedback by separating criticism or disagreement about a particular issue from any judgements of the person receiving the feedback. In other words, it is intended to make it easier for the recipient to hear and process criticism. Although it was once popular as a management tool in companies, it has since fallen out of favor with many people in the business world.

The popularity of the compliment sandwich at scientific meetings in 2022 reminded me of a different approach that used to prevail at seminars at the Marine Biological Laboratory in Woods Hole. Affectionately termed the “Monday Night Fights”, these events were characterized by wild and woolly discussions of data, methods and theories, during which some vocal audience members would vociferously disagree with points of view voiced by each other and the speaker. There were some who dreaded presenting in this venue. There were others who felt that if their new work would hold up under these conditions, it would pass muster anywhere. And there were members of the audience who went to these talks for their entertainment value, as well as for the scientific issues aired.

It is interesting to speculate whether the Monday Night Fights were better or worse for the progress of science than the compliment sandwich approach. For me, the more confrontational strategies of challenging speakers and ideas came easily: I grew up with a father with a loud voice who assumed he knew more than everyone around him about everything, so even as a young scientist I had no problem saying “you are wrong” to someone. Indeed, I much preferred out-front disagreements about ideas and positions to unspoken negative agendas, and have always found it hard to deal with scientists who hide their real opinions. On the other hand, I am starting to appreciate the potential utility of the compliment sandwich: lowering the psychological temperature around an issue may lead to less defensive conversations and give everyone an opportunity to articulate their points of view.

When I mentioned this to my sister, who is a literature professor and scholar , she pointed out that the compliment sandwich had dropped out of favor in business and other areas because many felt that it was ineffective for various reasons. First, it is human nature to focus on the praise and not fully hear the subsequent criticism. This is especially true if the criticism is well-buried in the middle of the sandwich. Second, some individuals may come to associate praise with coming criticism, thus sowing distrust of the praise, even when honestly meant. Third, to quote my sister: “compliments detract from the full-on engagement with the questions under discussion because they deflect attention away from the specific details of what is being discussed.”

But what then of seminars? Much of my own research has been influenced by questions and comments from seminar audiences. Some of those asking the questions and making the comments at these seminars were polite, but many were not, and some were downright hostile. However, the work in my lab has benefitted enormously from hostile challenges that forced us to think more deeply about our own work.

There is a fine line between being tactful and not being truthful. If we have learned nothing else from the past six years in politics, it is the importance of the truth, and how fragile our collective reality is when people lose the ability to hear the truth, and act on it. Clearly as scientists our goal must be the open and honest discussion of scientific disagreements. Of course, we should do our best to be collegial and fair, but we should not overlook flaws and shortcomings in scientific work. At the end of day, even if we opt for behavior akin to the compliment sandwich, it behooves us to ensure that the truth, as we see it, does not get lost in our efforts not to offend.

## Note

This essay is part of the Living Science collection.

**Figure 1:**
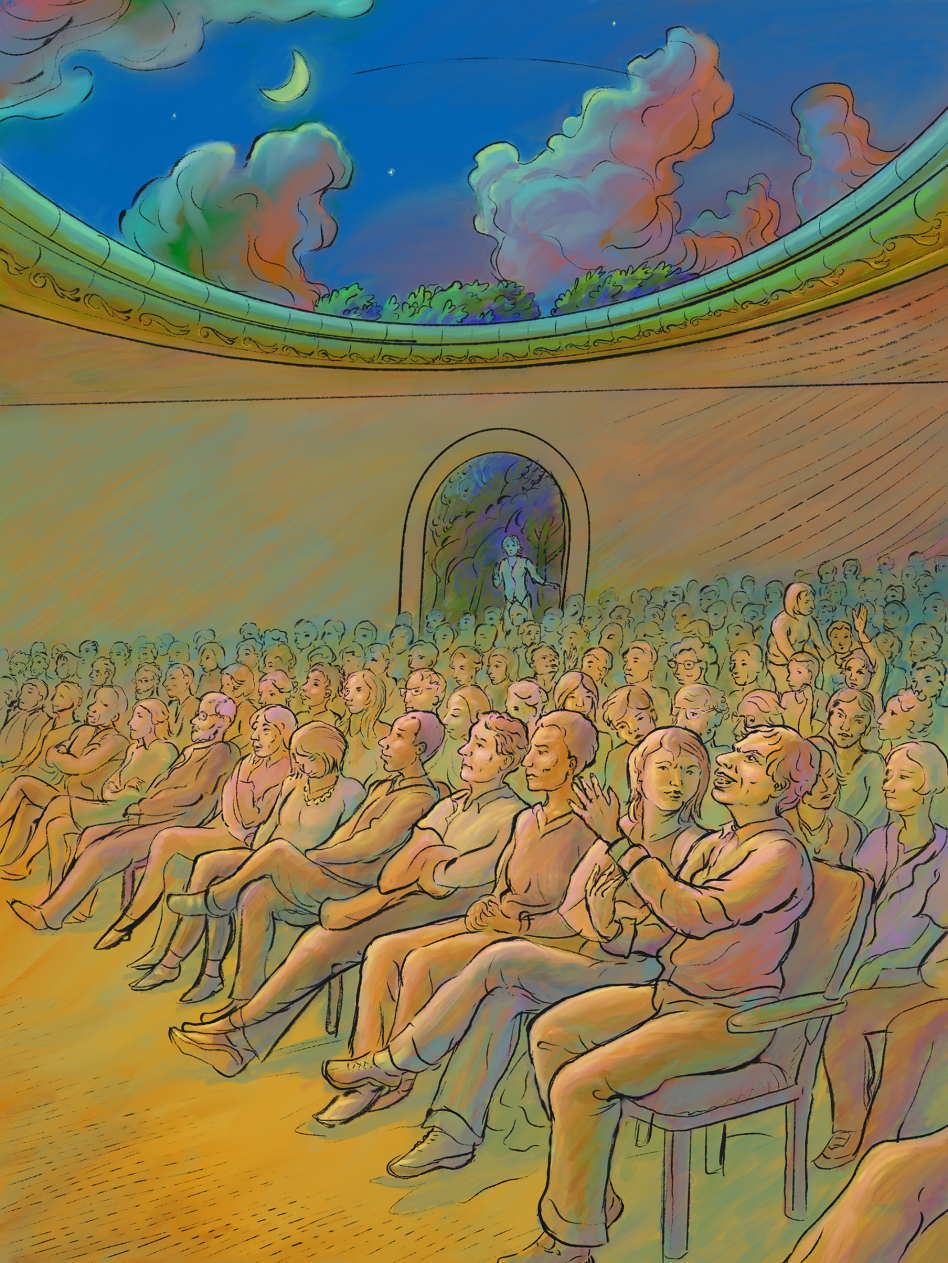
There are many ways to ask a question at a scientific meeting or seminar, and while we should do our best to be collegial and fair, we should not overlook flaws and shortcomings in scientific work.

Illustration: Ben Marder

